# Supplementary material for: MIC13-linked cristae disruption causes metabolic failure and early fibrotic remodelling in mitochondrial liver disease
Source: Cell Death Dis. 2026 Sep 15;17(1):801. doi: 10.1038/s41419-026-09283-y (PMC13578304; doi:10.1038/s41419-026-09283-y)

Western blotting
Figure 1

MIC13

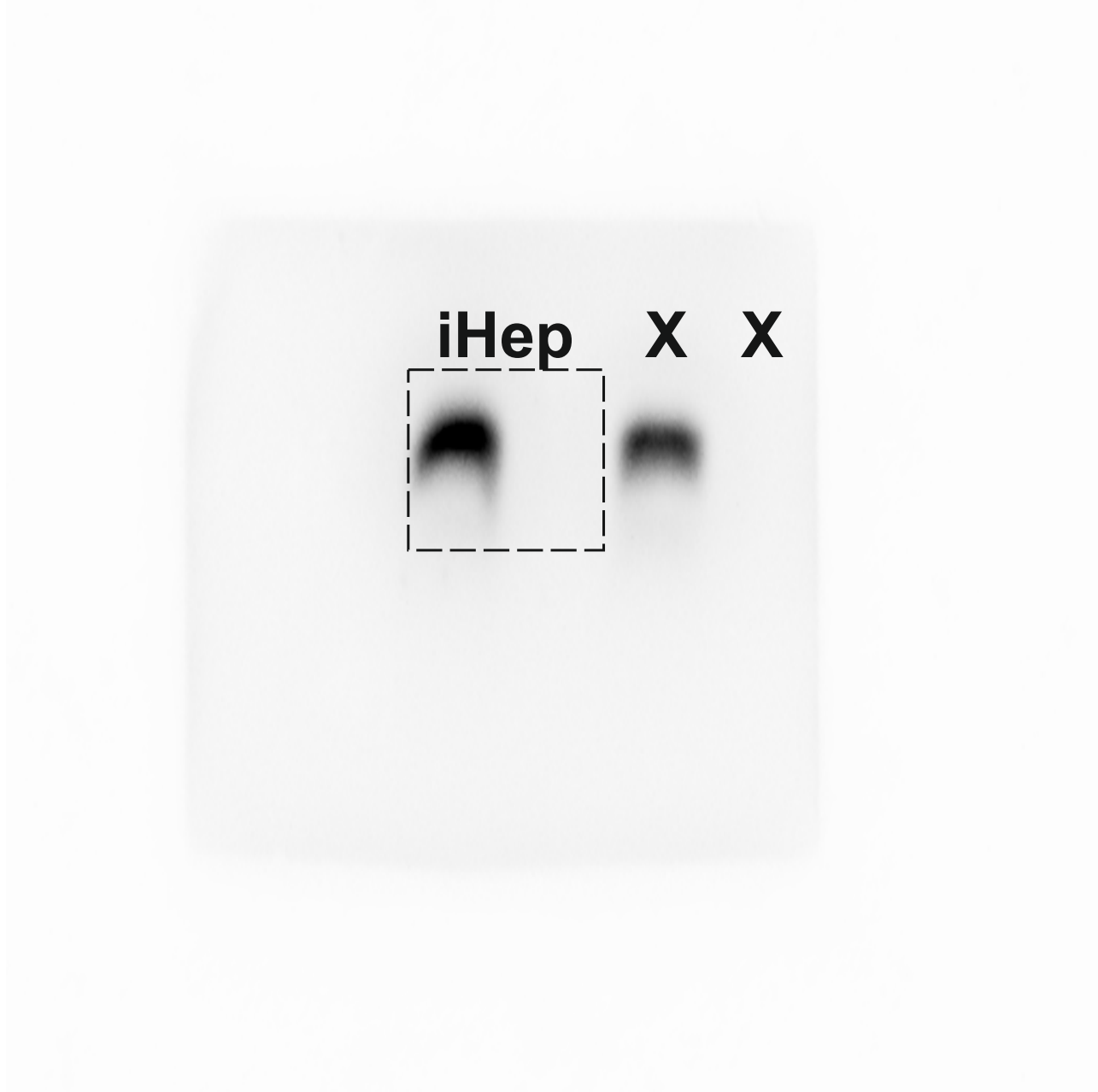

MIC26

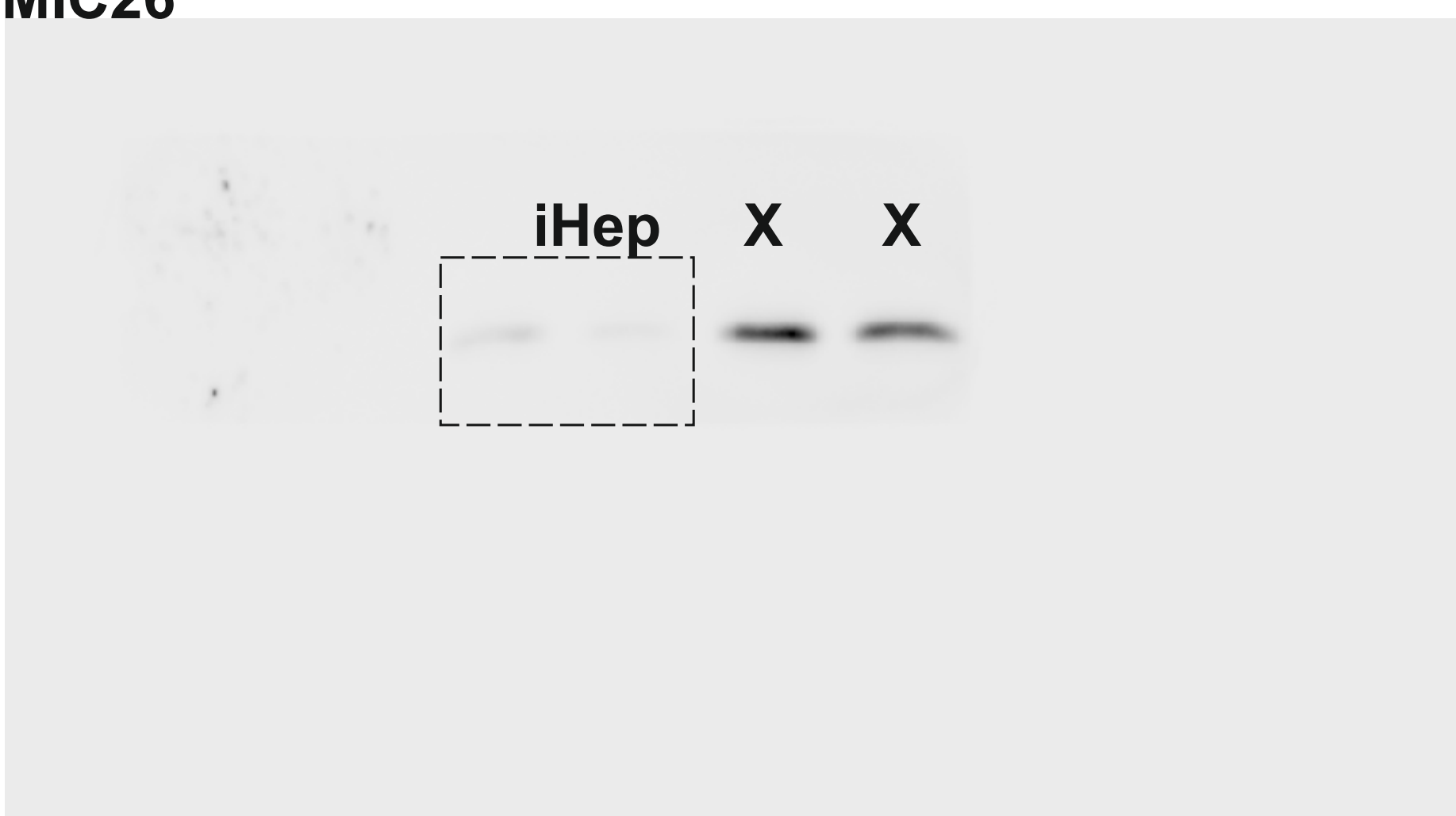

MIC27

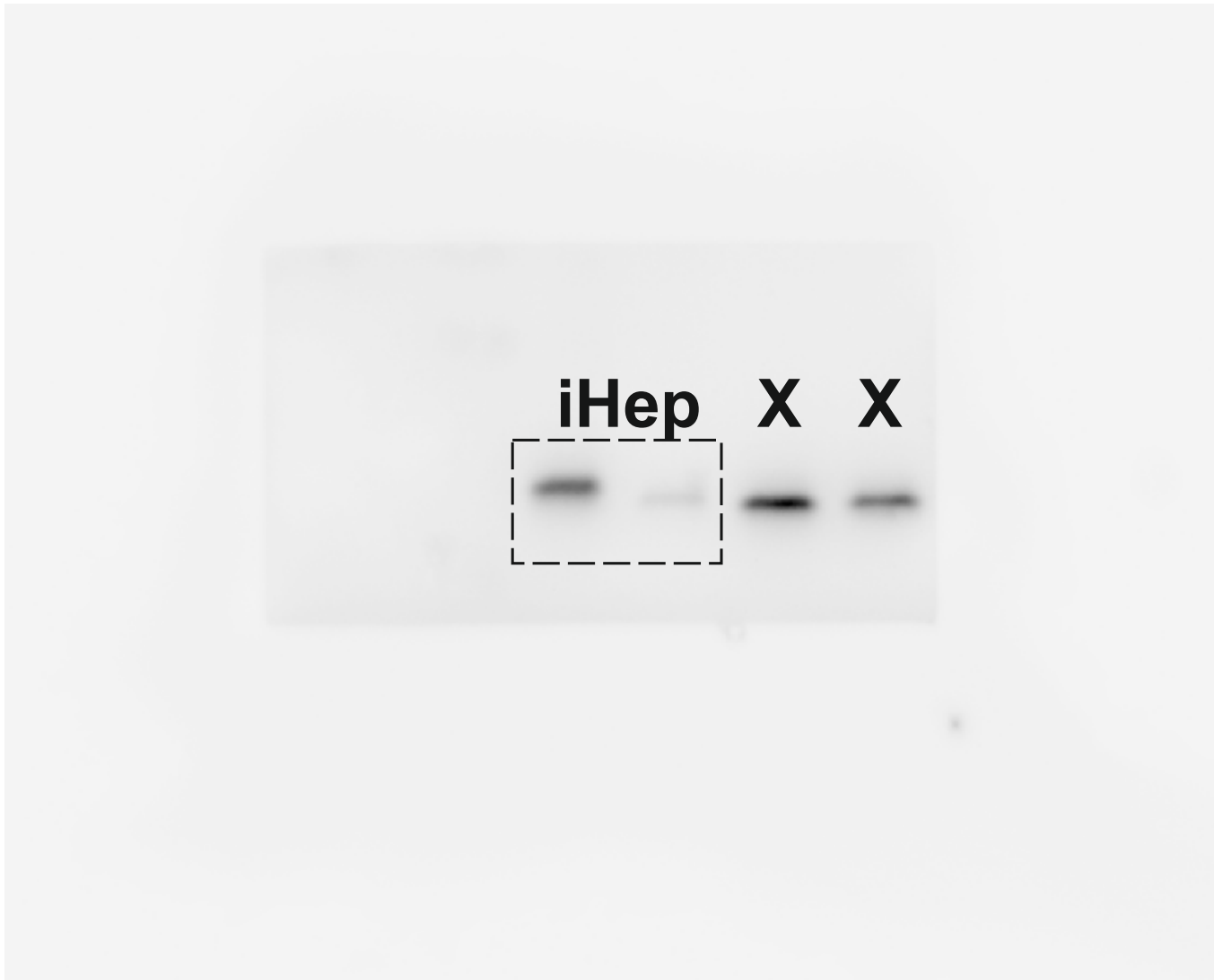

MIC10

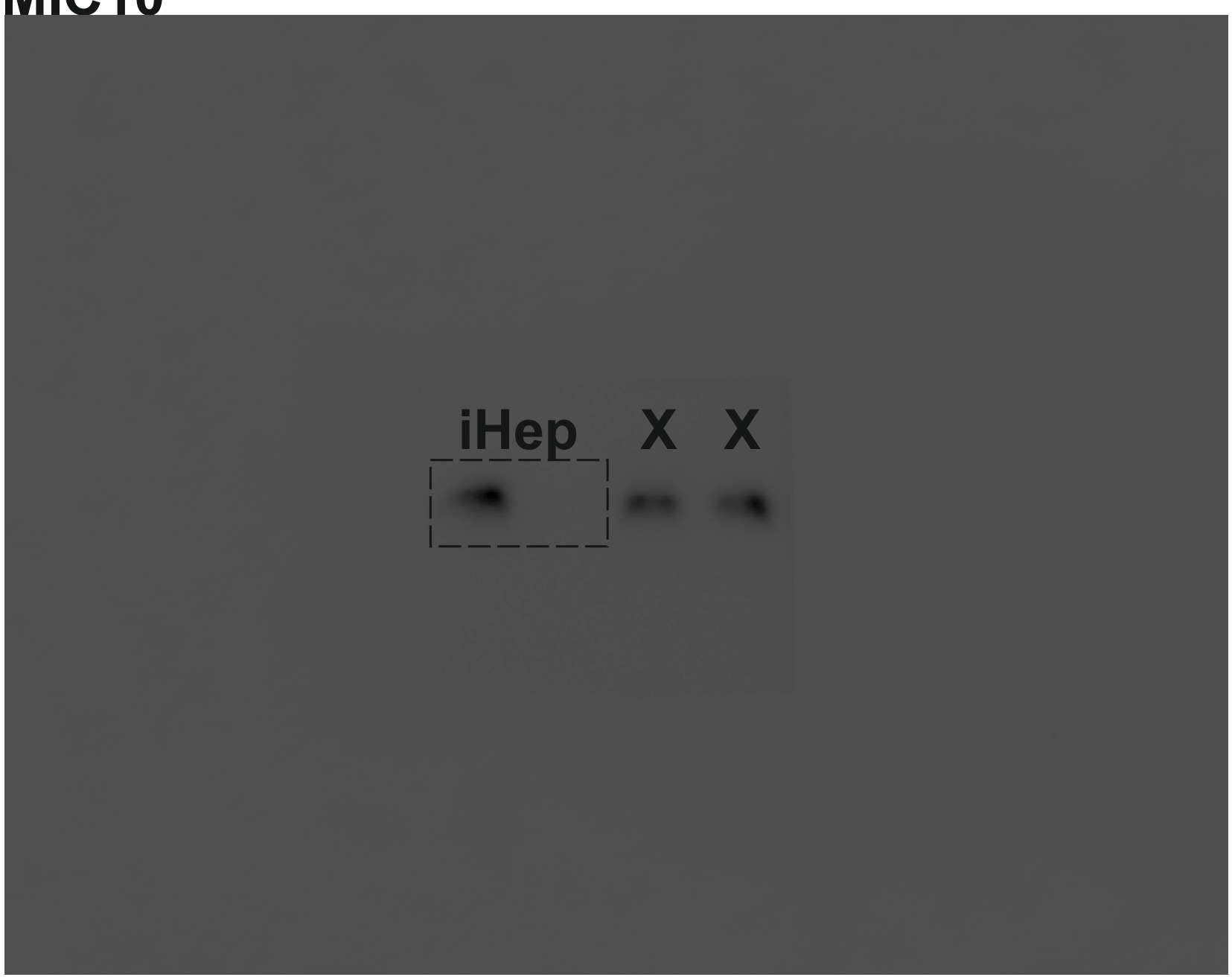

MIC25

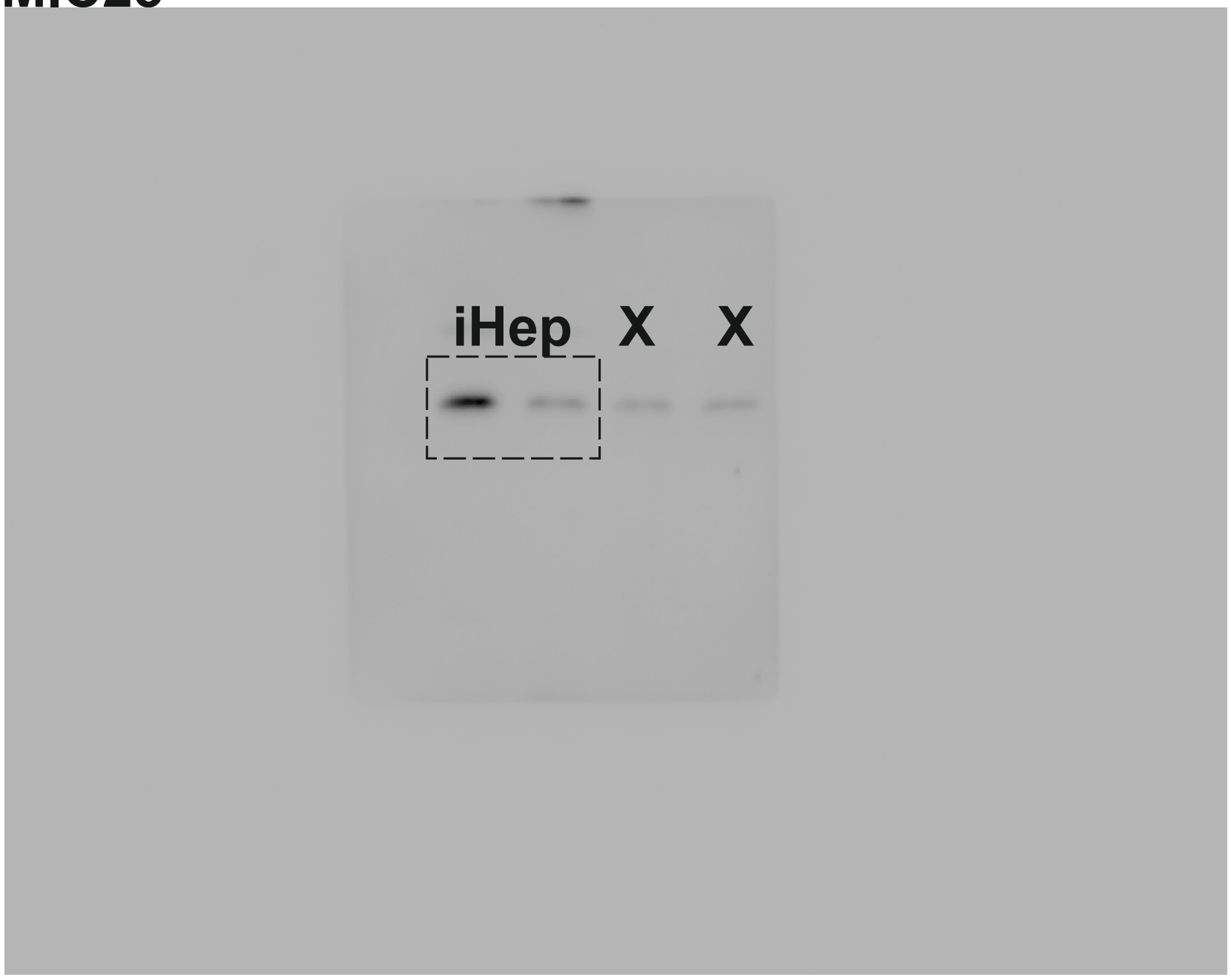

MIC60

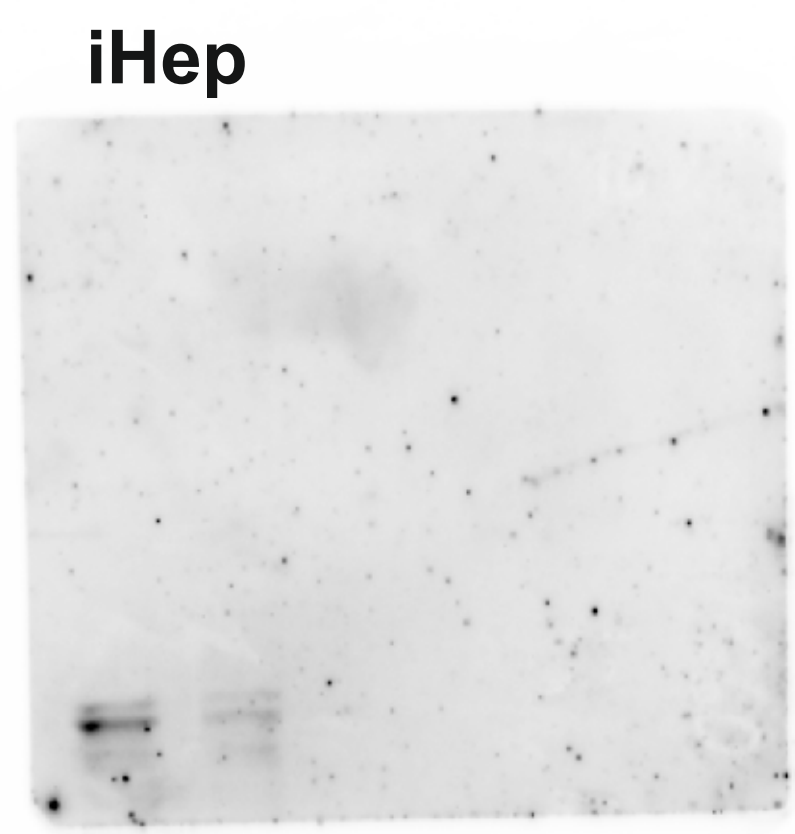

MIC19

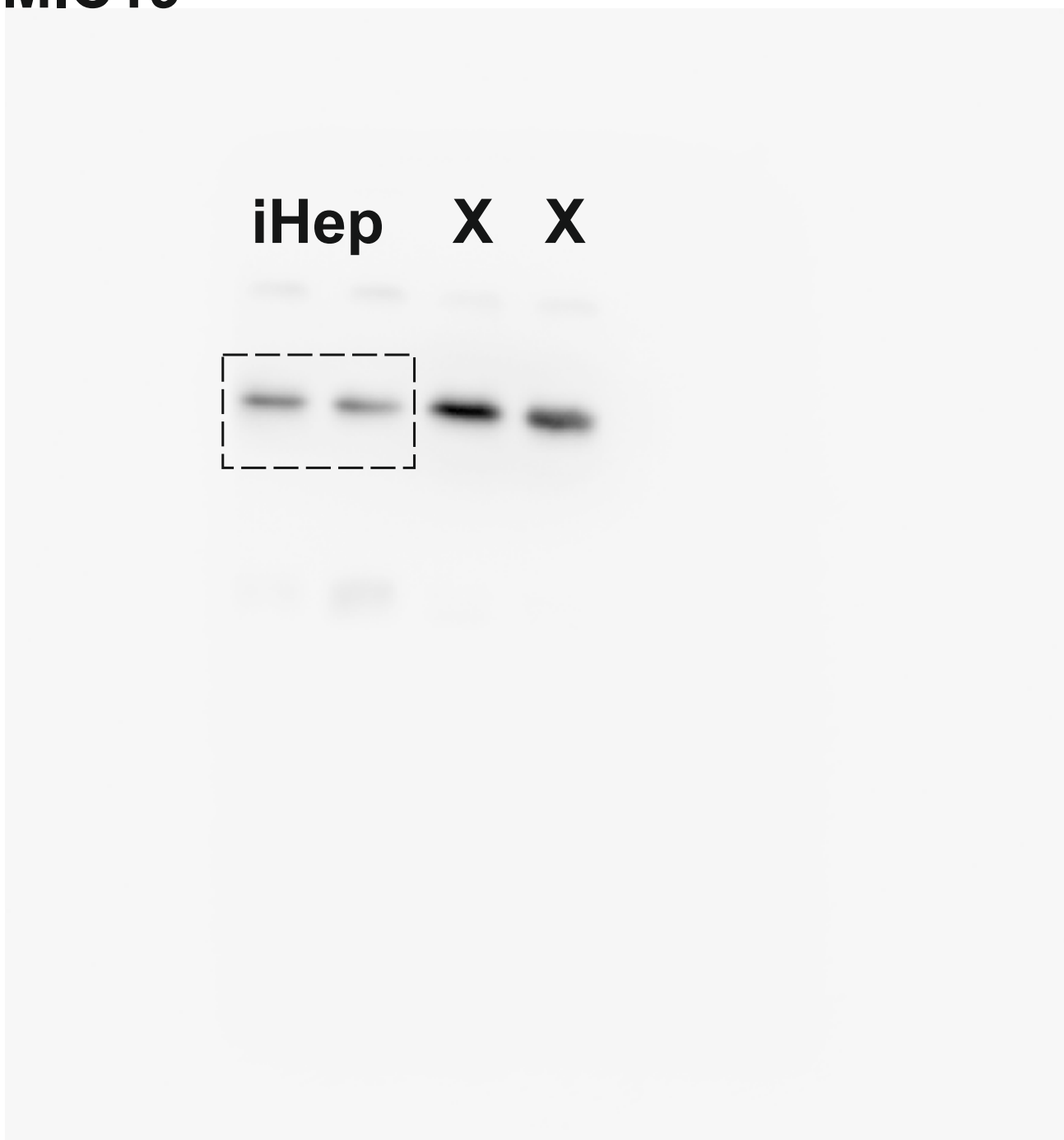

HSP60

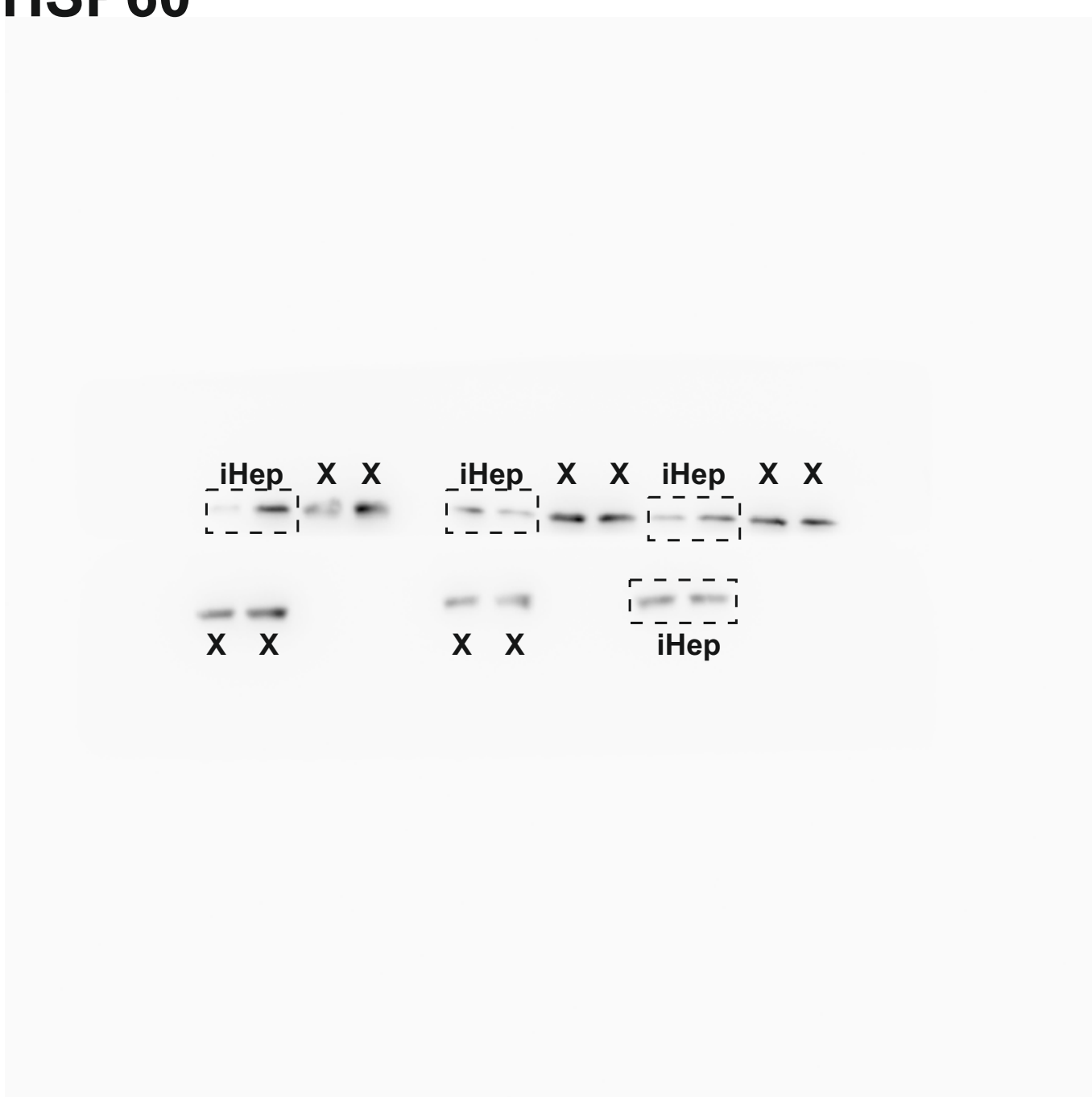

Supplement: Supplementary file 1 [file 41419_2026_9283_MOESM12_ESM.pdf]
