# Supplementary material for: MIC13-linked cristae disruption causes metabolic failure and early fibrotic remodelling in mitochondrial liver disease
Source: Cell Death Dis. 2026 Sep 15;17(1):801. doi: 10.1038/s41419-026-09283-y (PMC13578304; doi:10.1038/s41419-026-09283-y)
Supplement: Supplementary file 3 [file 41419_2026_9283_MOESM1_ESM.docx]

# Supplementary Information

## Case report

This boy was the fourth child of healthy, consanguineous parents with Iraqi ancestry. Three older siblings were healthy. Pregnancy was uneventful and the child was born without complications at 41+2 gestational weeks (weight 3300 g, lengths 55 cm, head circumference 34 cm, Apgar score 9/10/10, umbilical cord pH 7.17). On the second day of life, the neonate presented with hypoglycaemia (blood glucose 15 mg/dl, norm >45) and severe lactic acidosis (lactate level max. 16 mmol/l, norm <1.6; BE -23 mmol/l). A reduced quick value (28,1 %, norm > 55) was noted but liver values were normal. Metabolic work up revealed 3-methylglutaconic aciduria. Moreover, elevated levels of α-ketoglutarate and hydroxyphenolic acids were found as potential indicators of hepatopathy. Repeated measurements showed normal ammonia, creatinine and urea levels throughout the clinical course. Laboratory values improved with infusion and buffer therapy as well as adequate caloric supply. Genetic testing via exome sequencing revealed a homozygous splice variant c.260-2A > G in MIC13 as underlying disease pathology. The following clinical course was stable but the infant showed intermittent episodes with hypoglycaemia. At the age of two months, the child showed unclear episodes with cyanosis. At the time, EEG and cardiac work up were normal. At the age of 7 months, the child presented with severe liver dysfunction (GOT 1056 U/l, norm < 83; GPT 467 U/l, norm < 47; gGT 392 U/l, norm < 33; quick 59 %, norm > 78) and lactic acidosis (around 10 mmol/l, norm < 1.6). The child stabilized with supportive care and adequate caloric supply. However, at the age of 10 months another severe metabolic crisis occurred with transient respiratory insufficiency, epileptic encephalopathy and renal tubolopathy, which was triggered by a febrile viral infection. Brain MRI revealed global brain atrophy and symmetric basal ganglia lesions (Leigh-like syndrome). Although the child transiently stabilized, the clinical condition subsequently deteriorated. Finally, the child died at the age of 12 months due to progressive brain stem dysfunction and respiratory failure in a palliative care setting.

# Supplementary Figure legends

## Supplementary Figure 1: ROS levels and mtDNA release in MIC13^mut^ iHeps

(A) Intracellular ROS levels measured by DCFH-DA fluorescence showed no significant difference between MIC13^mut^ iHeps and WT, despite greater variability in mutant cells. Data is shown as violin plot with range, median and interquartile values indicated from 43 confluent fields of view per group across three independent experiments (n = 3). Statistical analysis was performed using Student’s *t* test. ns = non-significant, *p*-value >0.05.

(B) Quantification of extramitochondrial mtDNA puncta revealed no significant difference between WT and MIC13^mut^ iHeps. Data is represented as a violin plot indicating the range, median and interquartile values, with data from 50 cells from each group from three independent experiments. Statistical analysis was performed using Student’s *t* test. ns = non-significant, *p*-value >0.05.

## Supplementary Figure 2: Transcriptomics analysis of WT and MIC13^mut^ iPSCs and iHeps.

(A) Principal component scatter plot showing the separation between WT and MIC13^mut^ iPSCs and iHeps.

(B) Volcano plots of differentially expressed genes (DEGs), the selected genes from the GO terms categories including ECM organization, fatty acid metabolism, lipid metabolism are indicated.

## Supplementary Figure 3: Metabolic alterations in MIC13^mut^ iHeps

(A) Proteomics analysis reveals significantly altered proteins belonging to protein metabolism and autophagy, DPEP1 (dipeptidase 1), CPM (carboxypeptidase M) and SQSTM1 (sequestosome-1). Data collected from four biological replicates. Statistical analysis was performed by Student’s *t*-test with *: *p* value < 0.05.

(B) Expression of mitochondrial IDH2, IDH3 subunits, and ACO2 was not significantly altered in MIC13^mut^ iHeps.

(C) Urea concentration (mg/dl) in MIC13^mut^ iHeps and WT showed no significant difference between the group. Data are from three biological replicates (n = 3).

(D) Metabolomics data reveals significant changes in the SAM-dependent methylation metabolites, indicating impaired methylation in MIC13^mut^ iHeps. Data collected from five biological replicates. Statistical analysis was performed by Student’s *t*-test with *: *p* value < 0.05, ***: *p* value < 0.001.

(E) Bar graph representing the oxygen consumption rates (OCR), including BR (basal respiration), MR (maximal respiration), SRC (spare respiratory capacity), PL (protein leak), ATP prod (ATP production) and NMOC (non-mitochondrial respiration) from four independent Seahorse Analyzer runs, indicate a trend towards reduce respiration in MIC13^mut^ iHeps.

## Supplementary Figure 4: Alterations associated with lipid metabolism in MIC13^mut^ iHeps

(A) Metabolites related to phospholipid metabolism were significantly altered in MIC13^mut^ iHeps. Data collected from five biological replicates. Statistical analysis was performed by Student’s *t*-test with *: *p* value < 0.05, **: *p* value < 0.01, ***: *p* value < 0.001.

(B) Transcriptomics data analysis reveals significant changes in genes associated with fatty acid metabolism in MIC13^mut^ iHeps. Statistical analysis was performed by Student’s *t*-test with ***: *p* value < 0.001.

## Supplementary Figure 5: Cell-cycle progression in MIC13^mut^ iHeps using FACS-based assay.

(A) FACS-based analysis showed no significant difference in distribution of the of cells across different cell-cycle phases between WT and MIC13^mut^ iHeps (n = 3).

(B) Representative FACS gating profile used to determine cell-cycle phases in WT and MIC13^mut^ iHeps.

## Supplementary Figure 6: Transcriptional analysis of cell-cell adhesion genes in MIC13^mut^ iHeps

Heatmap showing DEGs associated with cadherins (CDH family), desmosomal cadherins, tight junctions and epithelial cell adhesion molecules (CAMs). Overall, these cell-cell adhesion genes were predominately downregulated in MIC13^mut^ iHeps.

## Supplementary Figure 7: Transcriptional analysis of inflammatory and innate immune pathways in MIC13^mut^ iHeps

(A) Gene set enrichment analysis (GSEA) of Hallmark pathway comparing WT and MIC13^mut^ iHeps identified inflammatory or innate immune pathways among the significantly downregulates, but not upregulated gene sets.

(B) Heatmap of genes associated with TNFα/NF-κB signalling demonstrated overall downregulation of key pathway components in MIC13^mut^ iHeps.

(C) Heatmap of genes associated with cGAS-STING / type 1 IFN pathway showed no evidence of pathway activation in MIC13^mut^ iHeps.

## Supplementary Figure 8: Metabolic regulation of ECM-associated remodelling pathways in MIC13^mut^ iHeps

(A) Metabolomic analysis showing significant increase in proline and glutamine levels in MIC13^mut^ iHeps, key metabolites linked to amino acid metabolism and ECM and collagen biosynthesis.

(B) Heatmap of DEGs involved in ECM-associated metabolic pathways, including α-ketoglutarate-dependent dioxygenases required for collagen post-translational modifications, including P3H2 (prolyl 3-hydroxylase 2), PLOD2 (procollagen-lysine,2-oxoglutarate 5-dioxygenase 2), and amino acid metabolic enzymes regulating glutamine and proline flux and α-ketoglutarate production, including GLS (glutaminase), ALDH4A1 (aldehyde dehydrogenase 4 family member A1), and OAT (ornithine aminotransferase). Together, these alterations indicate coordinated metabolic rewiring of amino acid pathways that converge on α-ketoglutarate-dependent collagen modification and ECM remodelling processes. **Supplementary Tables**

## Supplementary Table 1: Sequences of primers used in this study

| Gene | Strand | Sequence | Source Publication |
| --- | --- | --- | --- |
| RPS16 | fw | GCTATCCGTCAGTCCATCTCCAA | - PMID: 29990377 |
|  | rev | CCTTCTTGGAAGCCTCATCCAC |  |
| OCT4 | fw | CAGTGCCCGAAACCCACAC | - PMID: 29523187 |
|  | rev | GGAGACCCAGCAGCCTCAAA |  |
| SOX2 | fw | CCATGCAGGTTGACACCGTTG | - PMID: 29523187 |
|  | rev | TCGGCAGACTGATTCAAATAATACAG |  |
| NANOG | fw | TTTGTGGGCCTGAAGAAAACT | - PMID: 29523187 |
|  | rev | AGGGCTGTCCTGAATAAGCAG |  |
| SOX17 | fw | CGCACGGAATTTGAACAGTA | - PMID: 29466743 |
|  | rev | GGATCAGGGACCTGTCACAC |  |
| FOXA2 | fw | TTCAGGCCCGGCTAACTCTG | - PMID: 29990377 |
|  | rev | CCTTGCGTCTCTGCAACACC |  |
| HNF4α | fw | GTGCGGAAGAACCACATGTACTC | - PMID: 29990377 |
|  | rev | GAAGCATTTCTTGAGCCTGCAGTA |  |
| CEBPa | fw | GGTGGACAAGAACAGCAACGA | - PMID: 29990377 |
|  | rev | GTCATTGTCACTGGTCAGCTC |  |
| ALB | fw | AGCTGTTATGGATGATTTCGCAG | - PMID: 29990377 |
|  | rev | CCTCGGCAAAGCAGGTCTC |  |
| A1AT | fw | GGTCACAGAGGAGGCACCC | - PMID: 27308945 |
|  | rev | AGTCCCTTTCTCGTCGATGGT |  |
| AFP | fw | AGCAGCTTGGTGGTGGATGA | - PMID: 29990377 |
|  | rev | CCTGAGCTTGGCACAGATCCT |  |
| mtND1 | fw | CCACCCTTATCACAACACAAGA | - |
|  | rev | GGTTCGGTTGGTCTCTGCTA |  |
| RNR2 | fw | AACGATTAAAGTCCTACGTGATC | - |
|  | rev | TCCTTTCGTACAGGGAGGAAT |  |
| mtCYB | fw | CATCTTGCCCTTCATTATTGC | - |
|  | rev | GTTGTTTGATCCCGTTTCGT |  |

## Supplementary Table 2: Differentially altered metabolites in MIC13^mut^ iHeps

## Supplementary Table 3. Complete statistical results for all datasets
